# Chronic pain through COVID

**DOI:** 10.3389/fpain.2022.937652

**Published:** 2022-10-19

**Authors:** M. Dunham, L. Bacon, S. Cottom, P. McCrone, H. Mehrpouya, F. Spyridonis, T. Thompson, P. Schofield

**Affiliations:** ^1^ School of Health / Social Care, Edinburgh Napier University, Edinburgh, United Kingdom; ^2^ School of Design and Informatics, Abertay University, Dundee, United Kingdom; ^3^ Pain Association Scotland, Perth, United Kingdom; ^4^ Institute for Lifecourse Development, University of Greenwich, London, United Kingdom; ^5^ Department of Computer Science, Brunel University London, Uxbridge, United Kingdom; ^6^ School of Human Sciences, University of Greenwich, London, United Kingdom; ^7^ University of Plymouth, Plymouth, United Kingdom

**Keywords:** chronic pain, older people, COVID-19, remote healthcare, ageing

## Abstract

**Objectives:**

To identify good practice in the community management of chronic pain, and to understand the perspective of a group of healthcare service users towards the management of chronic pain using technology during the COVID-19 pandemic.

**Methods:**

Forty-five people, recruited *via* social media and Pain Association Scotland, participated in three focus groups hosted over Zoom. Focus groups were conducted using semi-structured questions to guide the conversation. Data were analysed using Ritchie / Spencer's Framework Analysis.

**Results:**

The participants shared observations of their experiences of remotely supported chronic pain services and insights into the potential for future chronic pain care provision. Experiences were in the majority positive with some describing their rapid engagement with technology during the COVID pandemic.

**Conclusion:**

Results suggest there is strong potential for telehealth to complement and support existing provision of pain management services.

## Introduction

Responses to the COVID-19 pandemic have highlighted the issue of service provision for people with chronic pain, especially older people and those living in remote communities (1). Services are being adapted and remodelled to meet the needs of service users^1^ and service providers in the midst of financial, structural and geographical constraints, with an accelerated move towards telemedicine as an alternative strategy for service delivery (2).

Older people live with a greater risk of developing both pain and dementia. For the very old population, living with Alzheimer's Disease and related dementias, the risk of experiencing manageable, yet unidentified pain is significantly increased (3). The challenging aspects of pain assessment in this population can also lead to under treatment (4).

Older people and those in remote or rural communities are acknowledged to have been disadvantaged when it comes to health and social care (5, 6). COVID has highlighted an existing problem, with people effectively stranded from face-to-face health care, but has also forced providers, carers and older people themselves to move more rapidly towards a range of alternatives to face to face health care. Recent survey by the UK Faculty of Pain Medicine (7) identified barriers during COVID including PPE shortages, remote working, shielding staff and new ways of working with a shortage of technology (8).

The focus of this research is the service-user's appreciation of technology's potential and usefulness in the support of chronic pain. Previous research on the assessment and management of chronic pain has largely excluded older people and is dominated by pharmaceutical approaches (9, 10). There is limited exploration of what technology means in terms of practical application, acceptability and feasibility for an ageing population (11). More specifically, the older person's own understandings and perceptions of the issue are marginalised in the literature.

There is some ambiguity in the taxonomy related to this topic. The terms telehealth, telecare, telemedicine, m-health, synchronous and asynchronous modalities are words and phrases amongst the new language of an evolving area of health care whose meaning and application is also rapidly changing with advances in technology (12). Lay and health professionals use and understanding of these terms appears to vary in the literature. For consistency of understanding, we will use the World Health Organisation's definition of telemedicine “the use of telecommunications and virtual technology to deliver health care outside a traditional health-care facility” (13).

As with much of health care research, this topic area is dominated by quantitative and positivist approaches which downplay the significance of individual experiences and personal understandings in favour of homogenous data sets. This study is intended to foreground development of larger research projects that are strengthened by their inclusive and participatory approach in their conceptualisation.

We explore adults' opinions on age-specific factors, with a focus on the older population, affecting adoption of technology and recommendations derived from their consultations. We consider the perspective of the service user to help inform and guide the development of a participatory approach to research suitable technology.

## Background

### Demographic ageing

The Western world is facing huge challenges in coming years. The World Health Organisation (WHO) has identified the ageing global population as an important medical and social challenge (14). As the global population increases the proportion of older people, those aged 80 and older, continues to increase. In 2015, the UK population over the age of 60 was 9 million and by 2050, it is anticipated that this population globally will double and exceed 2 billion (15). Worldwide, there are currently 145 million people aged over 80 years and by 2050 it is anticipated that there will be that many in China alone (16). By 2050 we will see 80% of the over 80 population living in low to middle income countries (15, 17). Rural communities in industrialised countries are in the main populated with older adults (18, 19). In the UK those aged 65 or over are now 11.4 million and are projected to grow by approximately 50% over the next 17 years (20). As the older population grows, chronic conditions like pain are more likely to develop and threaten independent functioning.

In the UK and Europe, we know that better health systems have improved life expectancy, but issues related to housing, geography and social deprivation continue to impact upon health and well-being, as we are seeing amongst the post-war baby boomers reaching their sixties (21, 22). However, living longer does not necessarily equate to better health. Despite a modern trend for reduction in smoking and alcohol consumption in rich countries (23, 24), smoking, alcohol use and inactivity, which all influence comorbidities, typically continue to affect older populations (25, 26). A number of comorbidities are seen in the older population including frailty, falls and cognitive decline. Whilst the WHO (15) does not highlight chronic pain amongst these syndromes, our recent systematic review (27) identified three specific pain syndromes in this population specifically back (16 studies), leg, knee or hip (16 studies), other joints (5 studies) and the updated version confirms these pain syndromes (10).

### Chronic pain

The International Association for the Study of Pain (IASP) defines chronic pain as that which persists beyond the expected healing time and suggests that it often has no identifiable cause, and it is often incurable (28, 29). The expectation being that the individual will have to learn to live with ongoing pain and this has resulted in the introduction of cognitive behavioural methods to support self-management of pain.

### Pain and ageing

Living with chronic pain is challenging. Access to pain services is often limited and for a short duration. We know that 40% of the older population living in the community have poorly controlled chronic pain (30, 31). The latest thinking in the field is towards self-management and consequently we have seen self-management programmes established around the UK in many pain services (32, 33). Older adults do not always or consistently have access to these programmes (31, 34). This is an issue which has been compounded by the recent COVID-19 pandemic, whereby many pain clinic staff have been relocated to frontline services. Or, in the case of the older population, they have been unable to access services due to isolating.

Pain services across the world have been severely disrupted by responses to the COVID-19 pandemic (1). The Faculty of Pain Medicine recently conducted a survey across the UK and found that 25% of pain services had stopped altogether during the pandemic with significant redeployment of staff (7). A second survey was carried out by the FPM and whilst they found that many functioning services had adapted to the constraints placed on them by the pandemic, such as PPE shortages, remote working, shielding staff and new ways of working with a shortage of technology (8). However, they also reported many problems associated with pain management programmes being provided online as a result of poorly functioning technology. It appears that throughout the pandemic, the only technology available for pain services was telephone or video. A recent paper by Eccleston et al. (1) suggested that there has been a move towards the introduction of telehealth and eHealth approaches in many areas, but this has been slow and often confounded by poor Internet access or equipment especially on the part of the service user.

### Technology

Technology use in health care, with potential to support the needs of older people with chronic pain, is increasing. Past work has shown that older adults are frequent users of technology (35). However, recent research has shown that whilst there's progress in use of eHealth solutions, this has been slow due to poor access to the Internet and technology on the part of the service user, an even more important issue for remote communities (36).

The types of digital health technologies and the terminology used to describe these is an evolving area (37). Most identified eHealth and mHealth tools for older people relate to health promotion and primary prevention including lifestyle programmes. The terms mHealth, eHealth, telemedicine and telehealth are often used interchangeably to describe the use of digital technologies, mobile and wireless devices, such as mobile phones, tablet computers, patient monitoring devices, and mobile applications (apps), to offer support for personalised care and the achievement of health care objectives (38). Some of the telehealth programmes have been designed to increase and promote activity or provide health information related to living with and managing particular diseases or chronic conditions.

The benefits of telehealth may arise from the sheer variety of options to enhance the quality of care across populations. Telehealth may be used to support initial assessment, interventions and education of people at home. It can also increase access to services to the disadvantaged, such as prison populations and remote communities, reducing unnecessary travel and associated costs. However, this may not be a suitable option for all. Evidence for the potential benefits of telemedicine's use is growing across a range of health areas (39). However, if telemedicine is to meet the needs of an ageing population it needs to be both reliable and acceptable. Much of the evidence for acceptability relates to the health professional's view. Recent survey data of Swiss older people referred to a local Pain Centre supports its use in chronic pain management (40). This anonymous voluntary survey noted a mean level of acceptance of telemedicine when expectations are met.

### Patient and public involvement (PPI)

In planning health care research, the importance of engaging with the service user, the patient, needs to be acknowledged. In the UK, national policy is driving for public involvement in research in the form of PPI (41). Establishing the relevant needs, experiences, fears and expectations for technology in a population is a valid research strategy. There is a growing movement for PPI contribution as an equal partnership in research development (42).

In terms of identifying the needs of the older population, Wethington et al. (43) facilitated a consensus workshop in Cornell University involving academics from the US and the UK. The workshop consisted of sixty participants including: older adults with pain and their caregivers, behavioural and social scientists, healthcare providers, pain experts, and specialists in mHealth and health policy. This workshop was designed to identify the research agenda for the use of technology by the older population with pain. and a number of recommendations were made (Table 1).

**Table 1:** Recommendations from consensus workshop.

*Conduct research on ways to enhance accessibility of mHealth tools among diverse groups of older adults with pain*
*Promote research/commercial partnerships and other initiatives that expedite bringing mHealth innovations into practice*
*Conduct research on the impacts of mHealth on physical and mental well-being*
*Expand research on mHealth sensing applications*
*Promote integration of users into basic research issues regarding mHealth and later-life pain*
*Conduct research on ways to personalize and tailor mHealth tools for individual users with pain*
*Expand research on ways mHealth data can inform intervention development and on ways to expand mHealth tool reach in clinical and non-clinical settings*
*Develop a core set of mHealth data and outcome assessments*
*Promote research on ways to initiate/sustain patient behavior change using mHealth tools*
*Conduct research on health system, workforce and patient education issues regarding mHealth use*
*Expand research on mHealth cyber-security and privacy issues*
*Expand research on sustainability of mHealth use at the patient, provider, and health system levels*
*Promote research on ways mHealth tools can improve patient-provider (and provider-provider) communication*

Wethington et al. (2018).

An earlier study by Philip et al. (44) explored the concept of technology and the impact upon personal and social interaction. Thus, examining the suggestion that technology use could replace the important personal contact which could be had by regular visits to or by the health care professional. The Technology for Older Adults: Maximising Personal and Social Interaction (TOPS) project examined interactions between rural older adults with chronic pain and their health and social care providers and considered how technology could play a part in enhancing life experiences (44). The project explored intersections between four themes, namely social isolation, chronic pain, health and social care and new (eHealth) technology. This project demonstrated that older adults across Scotland are receptive to technology for the management of their pain. Rural Scotland accounts for 98% of the landmass and 17% of the Scottish population, spread across many remote communities and islands and could be influential in this positive reception of technology (45). But it could be argued that such similar circumstances apply as a result of COVID-19.

### Study aim

This study is intended to inform further research. This project addresses the paucity of qualitative literature examining experiences of home-based chronic pain management and support. The purpose of this study was to identify good practice in the community management of chronic pain, with a focus on the older population. To understand the lived experiences of a group of healthcare service users towards the management of chronic pain using technology during the COVID-19 pandemic.

We aimed to address the following research questions: (i) How do community dwelling older people experience chronic pain programmes using telehealth technologies? and (ii) How acceptable are telehealth technologies to people living with chronic pain.

## Methods

These exploratory methods were informed by participatory approaches to co-design in research (46) and phenomenological methodology to understand the experiences of people living with chronic pain (47).

### Participants

Ethical approval for the study was obtained from Abertay University's Research Ethics Committee (EMS4573) and all interviewees provided consent for participation in the focus groups. Due to the pandemic and difficulties in arranging face to face meetings, the approach for recruitment in this exploratory study was an open invitation *via* social media. Participants self-identified as meeting the inclusion criteria, older adults living with chronic pain, accessed directly without any gatekeeper. Some of the participants were members of Pain Association Scotland, a national charity facilitating pain management education in the community, support but the majority were not known to each other. These factors may have influenced the numbers and type of participant. Although formal consent was an ethical requirement for participation, request for demographic details was an optional part of the consent form and this was not shared by all participants.

The participants were given an option to attend one of three focus groups facilitated by the research team *via* Zoom over a four-week period in 2021. The focus groups were majority female (40 female, 5 male).

### Data collection

Informed by a recent review of the literature (11) and the findings of Wethington's 2018 consensus workshop, the project team developed a semi-structured topic guide to explore the dimensions of experience and engagement with technology. The guide was used to inform the development of an interview protocol (Box 1) to conduct the focus groups. Specifically, the guide addressed (1) understanding of technology (2) access to pain services during COVID-19; (3) suitability of technology and innovations for managing pain in the future; and (4) privacy and security of data if such new technology were to be implemented.

Box 1 **Interview protocol.**

1. What do you understand to be technology and how do you use it in everyday life for the management of your pain?
2. How has your access to pain services being impacted with COVID? Have you been able to continue with GP consultations what are the services do you need to help you manage your pain?
3. Thinking about the use of technology what would you like to see to help you manage your pain in the future?
4. If we develop same technology and it did all the things you were talking about, how would you feel about your data going into the ether? Would you be happy with your information being shared with other health professionals?

Three focus groups were conducted between July and September 2021 *via* Zoom. Each group was facilitated by two members of the research team whilst two members of the team took notes. The Director of Pain Association Scotland was present to introduce the team. Each focus group consisted of 6–14 participants and lasted approximately 60 min. Ethical approval excluded contemporaneous digital recording. Note taking was undertaken by two members of the academic team to obtain contemporaneous records of conversations whilst two other team members facilitated the discussions, questions, and comments in the online chat facility.

### Data analysis

The transcribed data were examined using framework analysis to identify themes (48, 49). A deductive approach was adopted, *basing analysis on pre-existing theory*, that used the topic guide as an organising framework comprising of themes for the purpose of the coding process [(49), p. 3]. The coding was undertaken between the team independently and analysis was conducted by three experienced researchers (PS, MD / LB) comprising IT and health care research expertise. Following the stages of framework analysis, analysis commenced by familiarisation with the data. This stage involved initial annotation of the transcripts with notes and comments. The anonymised transcripts were read and re-read line by line starting by identifying a label or code to each particular sentence or section within the text. These initial codes were largely broad deductive codes, based on the overarching structured interview questions. The suitability of these codes was tested against one of the focus group transcripts by two of the authors and the code sheet was adapted after peer discussion. From this process of comparison and discussion amongst the team, substantive codes were expanded and applied iteratively throughout the transcripts then tabulated with exemplar quotes from the participants, collated and organised into emerging themes and sub-themes.

## Findings

Distinct themes emerged from within the areas of questioning including *discovery, activity, connecting and communicating, benefits, disadvantages and one-stop shop and inclusivity.* Notably, the dialogues centred around the novelty and sharing of experiences with both technology and living with pain itself.

### Understanding and current use of technology

The focus of discussion of the participants current engagement with technology was one of discovery. Some had significant experience of using technology and others had relatively little. However, in all three focus groups there were quite animated discussions of the variety and types of technology available to support living with a range of physical and psychological problems related to chronic pain, and just living.

#### Discovery

Some participants described how they used apps to plan their lives around the limitations of chronic pain. Web-based or phone facilities such as diaries, prompts and medication reminders were part of a range of newly discovered “helpful resources” to support planning and pacing. Many described using existing technology in new ways. These ways included use of household technology such as the Alexa facility of Amazon, and Fitbit technology. However, for a minority the potential for being overburdened with information available from internet sources was off-putting, as exemplified by this comment the “*sheer volume of information is overwhelming*”.

#### Apps for activity

Particular mobile phone apps were identified as helpful for supporting mental health, anxiety and general wellbeing. In all three focus groups there were detailed accounts of the ways they had engaged with Apps, Google and YouTube in searches for structured activity, advice regarding pacing, guided relaxation and similar. The potential use of Amazon's Alexa and other virtual assistants e.g., SIRI to prompt taking of medication and plan the day was recommended.

The sheer range of apps described was noteworthy, the identified apps included “MyFitnessPal”, “Headspace” and “Whitenoise”. The facility of symptom tracking alongside activity management was noted as helpful. Some apps were identified as useful for meditation and relaxation, others for exercises and support for activities. There had clearly been some experimentation, with some of the apps described as less helpful, such as not being free to use or the exercise suggestions being overambitious. One web-based exercise class run by an NHS physiotherapist was described as a “life saver”.

#### Connecting and communicating

In terms of communication with the outside world, participants found that technology helped them to maintain links with the outside world, although, some did mention that they did not like appearing on screen. The virtual world of online communities *via* hosting platforms such as Zoom, Houseparty, and Microsoft Teams had “*opened up new possibilities of finding people*” with the same health problems and reducing isolation.

The discovery of new online communities, including pain forums, the Pain Association Scotland group meetings, and the opportunity for peer support that these afforded was welcomed because “*someone always responds*”. For a few the possibility of being seen *via* a video interface was a cause of anxiety or perceived as less personal.

### Access to pain services during COVID-19

There were starkly contrasting experiences within the three focus groups. The methods of accessing GPs and chronic pain teams had dramatically changed from face-to-face meetings into phone or video calls *via* a range of platforms. Some had clearly found the new modes of communication a good experience and others were less “*smartphone savvy*”.

Descriptions of experiences of remote consultation were mixed; one participant had been offered physiotherapy remotely; this had helped them and they described a “*remarkable improvement*”. A participant from one of the Scottish islands exemplified the experience of some from rural communities. She shared her thoughts that the adaptations of service provision during COVID described how the rest of the world now understood her world, that is living remotely. Drawbacks included reliance on having a good internet connection, the equipment and ability to use it. One participant described a family member who would not use technology because of fear.

#### Benefits of remote pain support

Those participants who had access to support *via* pain association Scotland (PAS), had found the provision of webinar support positive and helpful with one person saying it was *a very good experience*. The participants were largely accepting of technology in their health care; (technology) “*changed my life and opened it up*”. For some, the use of technology was preferable to travelling and *reliable*. Two participants in focus group 3 described how their GPs, knowing they lived with chronic disease, had been proactive in contacting them regularly in the first “COVID lockdown” with one receiving regular video calls from the GP team.

#### Experienced disadvantages

Most found appointments, by whichever mode, difficult to obtain and there was some frustration expressed about not getting to see GPs regularly. The difficulty explaining a problem *via* a phone conversation was concerning, one participant felt “fobbed off” and said that it was “*hard to explain over the phone*”. Another participant described being passed from person to person and two said that their medication was not reviewed throughout (the pandemic). Another had paid to access private physiotherapy services in the lockdown. Fears of not being believed or being misdiagnosed because of not being seen were expressed by three of the participants.

### Technology and managing pain in the future

#### One stop shop

The possibilities of technology use had clearly grown for the participants. They identified a range of resources which they had adopted to help them plan their lives around chronic pain. These approaches included diaries, prompts and medication reminders which the participants described as helpful.

The possibility of freely accessible information in one place, having “*everything centralised*”, with clear evidence-based advice and support was a consistent request from each of the three focus groups. Access to PAS, peer support groups and self-management strategies all in one place was described as very important. Some asked if they could use technology and resources from this central point to directly support their pain management.

#### Connectivity and inclusivity

Access to online services was variable; for some remaining at home was preferrable to travelling and more reliable. Others reported that they found technology use hard, particularly over the phone where they “felt fobbed off” or that they were not mobile “savvy”. Having reliable and good quality high speed Internet access was acknowledged as a priority.

For some the possibility of combining face-to-face and Internet groups was an ambition. Participants described the importance of acknowledging and including the wider ageing populous and their possible communication needs; ensuring all website and apps were accessible to those with hearing impairment, sight loss, intellectual difficulty, language barriers or people who could not read English etc. Other ambitious suggestions included use of *voice activated* options and *text to speech* apps and similar. Cost of access, even nominal amounts, was also identified as a limiting factor with a clear consensus that apps and similar should be free at the point of use.

The importance of education technology presented in a more accessible form and just keeping up with changes as one person was exemplified

“ … *technology is a speedboat you need to get on or be left behind*.”

### Privacy and security with technology

The potential use of data and data protection was raised as an issue within all three focus groups. Only one person was concerned about consent. Some mentioned the need to consider anonymising any shared data. One person said they felt uncomfortable about “*their data”* being on the Internet. Data concerns highlighted were with who (non-health care professionals) could access data from any proposed app.

Out of all three focus groups, all hosted on Zoom, only one person was against the expanding use of technology in health care. This was a self-selecting group using technology for the focus groups which makes it not representative of the wider ageing population of the UK. However, it is noteworthy that the majority had embraced technology and were willing to explore a wide variety of possible future possibilities in the provision of chronic pain services and health care provision in general.

## Discussion

The aim of this study was to identify improvements to the design and delivery of remotely supported self-care for the management of chronic pain in remote/underserved communities. The use of technology was generally described as increasing significantly during the pandemic with most of the participants reflecting upon the pressure of the pandemic to force people to find new ways of accessing groups to maintain contact with their peers.

The use of technology, in the form of medical equipment, to manage certain conditions is not new. For example, Lehoux and colleagues (50) considered four different approaches to the delivery of healthcare using devices, to support home health care programmes. The approaches used were intravenous antibiotics, peritoneal dialysis, parental nutrition and oxygen therapy. They found that patients using these approaches were ambivalent about the drawbacks and advantages and found using them to be very restrictive and reduced social activity.

Technological approaches have advanced significantly in recent years with digital health tools, including mobile health applications (eHealth and mHealth) approaches, being used in many different settings (51). Their use reflects the range of approaches where current commonly used devices have been adapted for a medical/health promotion purpose to assist older people with independent living (52). Examples where devices have been used include to enhance medication adherence, a mobile application to support older people with oral anticoagulation treatment (53), support behavior change, by use of a wearable fitness tracker for older people with obesity (54), a text message facility and prompt to promote and increase exercise in older people (55), and support self-management of heart failure using a mHealth monitoring system and a health-related app (56).

In this study a few participants commented that they felt overburdened with the sheer volume of information. However, the participants in our earlier EOPIC study (57) found that the plethora of online resources for the management of pain was overwhelming, unreliable and constantly changing. Another study by Philip et al. (44) responded to the concerns previously raised that technology could reduce personal and social interaction, they looked across Scotland at this issue and were able to conclude that eHealth would be welcomed by patients and health care professionals due to the remoteness of the population, but it should not be at the expense of health and social care visits. The participants in our study also came from Scotland, so the geography is an important factor and of course, they had the added factor of being in “COVID-19 lockdown”.

Phone apps were described by our participants as being useful with apps for exercise, relaxation and meditation as being helpful. A study by Thurnheer et al. (58) reviewed 15 papers where 1962 patients confirmed our findings in that patients find pain apps very helful in managing their pain, particularly for those in the community. Although, they cautioned the need for more scientific investigation.

The COVID-19 pandemic caused a major impact upon all NHS services, but pain services were hit particularly hard, with pain services suspended when some pain teams were relocated into high dependency areas to support the influx of COVID-19 patients. This was very unfortunate for many given that international human rights law guarantees the fundamental right to access to pain management (59). There is now emerging evidence that long-COVID may consequently present as chronic pain (60), thus adding significant burden onto already struggling pain services. Furthermore, chronic pain patients may be significantly impacted by COVID-19 infections as a vulnerable group when many live with co-morbidity (1).

Use of online social networks has been identified as providing opportunities to promote healthy behaviour and enhanced quality of life (61) however it is noteworthy that none of the participants mentioned use of Facebook or similar social media platforms.

Having strong reliable Internet access is essential and sadly, not something that is widely available to all.

Previous research has demonstrated some of the issues related to the use of technology by the older population relate to cost, unreliability, attitudes and mistrust (62), all of which were also identified by our participants. However, research has demonstrated that the fastest growing “users” of the Internet is the older population (63). It is important however, as highlighted within our study, that education is the key and that the technology is provided in an accessible format.

Concerns about using technology or participating in online communities were minimal. A recent systematic review of barriers and facilitators by Wilson et al. (64) in Australia identified 14 papers which discussed barriers and facilitators. Of these only three studies identified participants who were concerned about privacy and confidentiality. These were studies related to mental health. However, the participants clarified this by confirming that if the technology was developed by health professionals, they would trust it and the data collected.

### Limitations

There are limitations with this study. The main weaknesses of our study may be found in its homogeneity. This was a group of adults who self-selected to participate in the study, the majority of whom came from one organisation, which is not representative of the older pain population. Some of the focus group members (three people) had the advantage of being known to each other, one wonders whether this would have been more challenging for participants who were strangers. The three focus groups had mainly female participants, this could reflect a higher prevalence of chronic pain in women or more women accessing support to manage their chronic pain.

Most of the focus group members had already been participating in various Zoom meetings during the COVID-19 lockdown period and a small number of the participants were already known to each other through Pain Association Scotland which resulted in a relationship already present. Furthermore, the pandemic has forced people to consider alternative approaches to get support.

Pain Association Scotland and other pre-established health support groups had moved their existing meetings from face-to-face to online in order to maintain their peer support mechanisms. Therefore, these focus groups could be viewed as an extension of that peer support. Regarding data security, it is also possible that this small group of individuals were not aware of the growing potential for abuse of data.

## Conclusion and recommendations

The information collected in our three focus groups is supportive of the studies previously reported in the literature which demonstrate that older adults with chronic pain are happy to look at alternative methods to delivery of their pain support networks. The participants in this study generally wanted to use more technology, resistance was minimal with costs the main barrier. The participants were hungry for more technological advance, in the broadest sense. However, more research is required with larger more heterogenous populations of older adults, not already part of an established group, along with more information on the types and accessibility of technology. This could lead to the co-design of technology in the future.

Finally, our research identified a number of recommendations for future work and for the design and delivery of remotely supported self-care for the management of chronic pain in remote/underserved communities, presented below under our four topic areas:

1. **Understanding and current use of technology**
  1.1. Make use of existing household technology (e.g., Amazon Alexa or wearables such as Fitbit) to support self-management.
  1.2. Mobile phone apps should be considered for supporting general wellbeing, including mental health, anxiety, symptom tracking, activity management, as well as for meditation, relaxation and exercise.
  1.3. Social isolation can be reduced through the use of online communities (e.g., Zoom, Houseparty, Microsoft Teams) and peer support from people with similar health issues.
  1.4. However, be mindful of “information overload” which can be off-putting for older users.
2. **Access to pain services during COVID-19**
  2.1. Consider remote pain support through facilities such as webinars that offer more interactivity and presence compared to phone consultations alone.
  2.2. Offer therapeutic interventions remotely, where possible.
3. **Technology and managing pain in the future**
  3.1. Centralised and free access to services is an advantage, including access to PAS, peer support groups and self-management strategies, and should be considered, where possible.
  3.2. Prioritise reliable and good quality high speed Internet access to enable the above.
  3.3. Offer a combination of in-person and online support.
  3.4. Ensure that all websites and apps are accessible to those with hearing impairments, sight loss, intellectual difficulty, language barriers or people whose English is not their first language.
  3.5. Consider offering use of voice activated options and text to speech apps or similar.
4. **Privacy and security with technology**
  4.1. Compliance with Data Protection laws and policies is essential to mitigate any concerns with shared personal and sensitive data.

## Data Availability

The datasets presented in this article are not readily available because the data is confidential and anonymous. Requests to access the datasets should be directed to m.dunham@napier.ac.uk.

## Footnotes

^1^Throughout this paper the terms patient and ‘service-user’ are used interchangeably
